# The potential role of *Escherichia coli* as an indicator of environmental antimicrobial resistance in an urban river in Japan

**DOI:** 10.1038/s41598-026-56659-3

**Published:** 2026-06-28

**Authors:** Kazuaki Matsui, Rahman Md Mizanur, Tsuguri Inui, Mizuki Oda, Takeshi Miki

**Affiliations:** 1 Department of Civil and Environmental Engineering, Kindai University, 3-4-1 Kowakae, Higashiosaka, 577-8502 Osaka Japan; 2 Graduate School of Science and Engineering, Kindai University, 3-4-1 Kowakae, Higashi-Osaka, 577-8502 Osaka Japan; 3 Research Institute for Science and Technology, Kindai University, 3-4-1 Kowakae, Higashi-Osaka, 577-8502 Osaka Japan; 4 Faculty of Advanced Science and Technology, Ryukoku University, Otsu, Shiga Japan; 5 Center for Biodiversity Science, Ryukoku University, Otsu, Shiga Japan

**Keywords:** Antimicrobial resistance, CHROMagar^™^ ECC, Coliform, Combined sewer overflow, *Escherichia coli*, Environmental sciences, Microbiology

## Abstract

Coliforms, including *Escherichia coli*, are widely used as fecal indicator bacteria (FIB) in water quality assessments. However, the potential of these bacteria to act as reservoirs of antimicrobial resistance (AMR) in urban rivers under wet-weather conditions potentially influenced by combined sewer overflow (CSO) discharge remains insufficiently characterized. Using a One Health perspective, we evaluated temporal and weather-related variation in the concentrations of *Escherichia coli* and non-*E. coli* coliforms and in the proportions of colonies exhibiting ampicillin resistance or intermediate tetracycline resistance in the Hirano River, an urban river in Osaka, Japan, between October and November 2023. Sampling was conducted 10 times between October 17 and November 14, 2023, including sampling conducted 33 and 15 h after strong rainfall events classified as having the potential to trigger CSOs. Samples were analyzed using CHROMagar™ ECC medium for bacterial enumeration and to test resistance to ampicillin and tetracycline. Both non-*E. coli* coliform and *E. coli* concentrations increased by more than two orders of magnitude in the sample collected 15 h after a strong rainfall event classified as having the potential to trigger a CSO, suggesting possible contributions from CSO-related inputs and/or other fecal contamination sources. *E. coli* exhibited higher resistance ratios, with averages of 23.1% for ampicillin resistance and 12.3% for intermediate tetracycline resistance, compared with 3.7% and 0.8%, respectively, in the non-*E. coli* coliform fraction, supporting the potential utility of *E. coli* as an indicator of antibiotic resistance among culturable coliforms in urban river water. These findings support the potential utility of *E. coli* for both fecal contamination assessment and environmental AMR surveillance in urban river systems.

**Supplementary Information:**

The online version contains supplementary material available at10.1038/s41598-026-56659-3.

## Introduction

Waterborne pathogens are introduced into aquatic environments primarily through fecal contamination^1,2^. To evaluate fecal contamination and assess water safety, culture-based detection of fecal indicator bacteria (FIB) has been widely used^3,4^. Historically, the abundance of coliforms in aquatic environments has been considered an indicator of fecal contamination, but their reliability as proxies for pathogenic microorganisms is limited by environmental factors and non-fecal sources^5^. Because some coliforms can be found naturally in soil and water, they are considered less-specific indicators of fecal contamination^6^. With the development of differential and selective media for fecal indicator species such as *Escherichia coli*, the enumeration of *E*. *coli* has become the standard method for assessing fecal contamination, offering greater specificity and reliability than total coliform counts^7^. Although the gut is the primary habitat of *E. coli*, it can persist for a long time in secondary environments such as soil, water, and sediment. Its environmental persistence and naturalization were highlighted in the recent reviews^8,9^ and were further supported by primary studies that demonstrated *E. coli* survival and growth in non-host habitats^10,11^.

It is important to detect and quantify *E*. *coli* and other fecal coliforms not only as indicators of the risks to human health posed by co-occurring enteric pathogens, including virulent coliform strains, but also because of their role as potential carriers of antibiotic resistance genes^12^. Both pathogenic and non-pathogenic bacteria can acquire antimicrobial resistance (AMR), and non-pathogenic environmental bacteria may serve as reservoirs of resistance determinants potentially transferable to clinically relevant bacteria^13^. Recognizing this, the World Health Organization (WHO) has emphasized the need to understand the environmental dynamics of AMR within the “One Health” framework^14^. However, the behavior and distribution of antibiotic-resistant bacteria in aquatic environments remain poorly characterized^15^.

Clinical surveillance data from Japan for the period 2014 to 2023 indicated that the annual prevalence of ampicillin resistance in *E. coli* ranged from 49.2–52.6%^15,16^. Comparable resistance levels have been observed for other coliforms, with rates of 77.4–80.5% in *Klebsiella pneumoniae* and 79.0–82.9% in *Enterobacter cloacae*^15^. The notably high resistance rates observed in clinical isolates of *K. pneumoniae* and *E. cloacae* underscore the importance of monitoring not only *E. coli* but also other coliforms as potential AMR reservoirs. Although clinical surveillance data for tetracycline are limited, surveillance data from Japan spanning 2013 to 2022 in diseased food-producing animals indicated that the annual prevalences of tetracycline resistance in *E. coli* were 47.5–62.4% for swine and 43.0–62.5% for chickens, whereas those of ampicillin resistance in *E. coli* were 26.0–44.1% for swine and 23.9–43.5% for chickens^17,18^.

Although AMR is a growing global concern, research in aquatic environments has disproportionately focused on *E. coli*, with far fewer studies examining antibiotic-resistant coliforms more broadly. Characterizing resistance patterns among coliforms in river systems is essential for understanding environmental AMR dynamics within the One Health framework.

In this study, using a One Health perspective, we investigated temporal and weather-related variation in the concentrations of *E. coli* and non-*E. coli* coliforms and in the proportions of colonies exhibiting ampicillin resistance or intermediate tetracycline resistance in the Hirano River, an urban river in Osaka, Japan, during October–November 2023. Using chromogenic agar medium, we differentiated *E. coli* colonies from non-*E. coli* coliform colonies while simultaneously estimating their resistance ratios. To establish baseline coliform counts, sampling was conducted every 3 days for one month. Sampling conducted after strong rainfall events classified as having the potential to trigger CSOs enabled us to examine wet-weather changes in the concentrations and resistance ratios of *E. coli* and non-*E. coli* coliforms under conditions potentially influenced by CSO-related inputs. On-site measurements of water temperature, pH, conductivity, and dissolved oxygen (DO) were also conducted to provide water quality context for interpreting changes in *E. coli* and non-*E. coli* coliform concentrations after rainfall events.

## Methods and materials

Sampling was conducted between October 17 and November 14, 2023 at a bridge over the Hirano River (34.63530° N, 135.54944° E), an urban waterway that flows through a densely populated urban area of Osaka City, Japan (Fig. S1). A CSO discharge outlet is located 100 m upstream of the sampling point. Meteorological data, including total precipitation, maximum intensity, and duration, were obtained from the Japan Meteorological Agency (https://www.jma.go.jp/jma/). To classify rainfall events, we used the rainfall intensity index previously applied by Matsui and Miki^19^, calculated as (Total precipitation [mm] × Maximum intensity [mm h^−1^]/Duration [h]). In accordance with that previous study, unrounded index values exceeding 10 were classified as strong rainfall events. In the present study, this classification was used only to identify rainfall events with the potential to trigger CSOs; it was not used as direct evidence that CSO discharge occurred or was ongoing at the time of sampling. The meteorological observation station was located approximately 6 km from the sampling site. On-site measurements of water temperature and pH were conducted using a pH meter (D-71, Horiba, Kyoto, Japan), while conductivity and DO levels were measured using a multiparameter meter (Multi 3420, WTW, Bremen, Germany). The bucket was cleaned prior to sampling following an environmental DNA sampling protocol^20^. Briefly, a sodium hypochlorite foam cleaner (3.0%) was sprayed onto the inner surface of the bucket and the tip of the rope. After a few minutes, both were thoroughly rinsed with tap water and air-dried for 2 days before sampling. Sodium thiosulfate was not used because the extended air-drying period after rinsing was considered sufficient to allow residual chlorine to dissipate. Using a cleaned bucket attached to a rope, river water samples were collected from a depth of 10–30 cm below the surface on every third day for 1 month (October 17, 20, 23, 26, and 29, 2023, and November 1, 4, 8, 11, and 14, 2023, except for the interval from November 4 to 8), for a total of 10 samples. Sampling was performed at 11:00 a.m., and the collected samples were immediately stored in a cooler box with ice packs and transported to the laboratory. All experimental procedures were initiated within 6 h of sampling.

The culturable concentrations of *E. coli* and non-*E. coli* coliforms were quantified as colony-forming units (CFUs) using CHROMagar™ ECC chromogenic agar medium (Kanto Chemical, Tokyo, Japan). The same procedure was applied to antibiotic-resistant *E. coli* and non-*E. coli* coliforms, but with the medium supplemented with 50 µg/mL of ampicillin or 10 µg/mL of tetracycline. These concentrations were used to distinguish resistant populations from sensitive ones; therefore, no individual strains were isolated for downstream antimicrobial susceptibility testing (AST). The clinical minimum inhibitory concentration (MIC) breakpoints are 32 µg/mL for ampicillin and 16 µg/mL for tetracycline^21^. Resistance to ampicillin was defined as growth at concentrations above the clinical breakpoint (50 µg/mL), indicating full resistance. In contrast, growth on tetracycline-supplemented medium (10 µg/mL), which is below the clinical breakpoint, was interpreted as intermediate resistance. Similar thresholds were used in a previous study^22^.

Water samples were processed following ISO 9308-1:2014 guidelines^23^. Water was filtered through 0.45-µm pore-size mixed cellulose ester filters (Advantec, Tokyo, Japan), which were then placed directly onto CHROMagar™ ECC agar plates and incubated at 37 °C for 24 h. Colonies were differentiated according to the manufacturer’s specifications: blue colonies were identified as *E. coli*, whereas mauve colonies were identified as non-*E. coli* coliforms. In the present study, the mauve colony group is therefore referred to as the non-*E. coli* coliform fraction. This differentiation is based on enzymatic activity: β-glucuronidase in *E*. *coli* cleaves a chromogenic substrate to produce a blue/blue-green color, while β-galactosidase in non-*E. coli* coliforms cleaves a separate substrate to yield a pink/red color^3^.

To ensure that colony counts fell within the quantifiable range of 20–200 CFUs per plate, a series of filtered volumes (0.01, 0.05, 0.1, 0.5, 1, 5, 10, 50, 100, 250, and 500 mL) was used for each sample. For volumes of less than 10 mL, 10 mL phosphate-buffered saline (PBS) was added to the filtration funnel and mixed prior to filtration. The membrane was filtered with only 10 mL of PBS, which was used as a negative control to confirm the absence of colony formation. The PBS and all filtration equipment were autoclaved before the experiments. Each sample was analyzed in triplicate, and the results were expressed as CFUs per 100 mL. For each bacterial group and sampling date, mean CFU values were calculated separately from three technical replicates on unsupplemented medium and on medium supplemented with either ampicillin or tetracycline. The ampicillin resistance ratio and intermediate tetracycline resistance ratio were calculated as the mean CFU value on the corresponding antibiotic-supplemented medium divided by the mean CFU value on unsupplemented medium for the same bacterial group, multiplied by 100. Because colony counts on unsupplemented and antibiotic-supplemented media were not paired at the replicate level, variability was not propagated to these ratios; therefore, error bars are not shown.

All statistical analyses were performed using R software^24^. Spearman rank correlation analysis was conducted using the “cor()” function to evaluate monotonic relationships between environmental parameters and bacterial abundance (CFUs). Correlation matrices were visualized using the *ggcorrplot* v0.1.4.1 package^25^, with numeric labels indicating Spearman correlation coefficients. Due to the limited sample size (*n* = 10), statistical significance testing was not performed for the Spearman correlation analysis, and only correlation coefficients are reported. Red and blue were used to indicate positive and negative correlations, respectively. Differences in *E*. *coli* and non-*E. coli* coliform concentrations were assessed using a generalized linear mixed-effects model (GLMM) implemented via the “lmer() function” in the *lme4* package^26^, with bacterial type (*E*. *coli* or non-*E. coli* coliform) treated as a fixed effect and sampling date as a random intercept. CFU data were log_10_-transformed prior to statistical analysis to improve normality and stabilize variance. Significance was assessed at an alpha level of 0.05. For the GLMM analysis, *p* values were obtained using the *lmerTest* package.

## Results and discussion

**Fig. 1:**
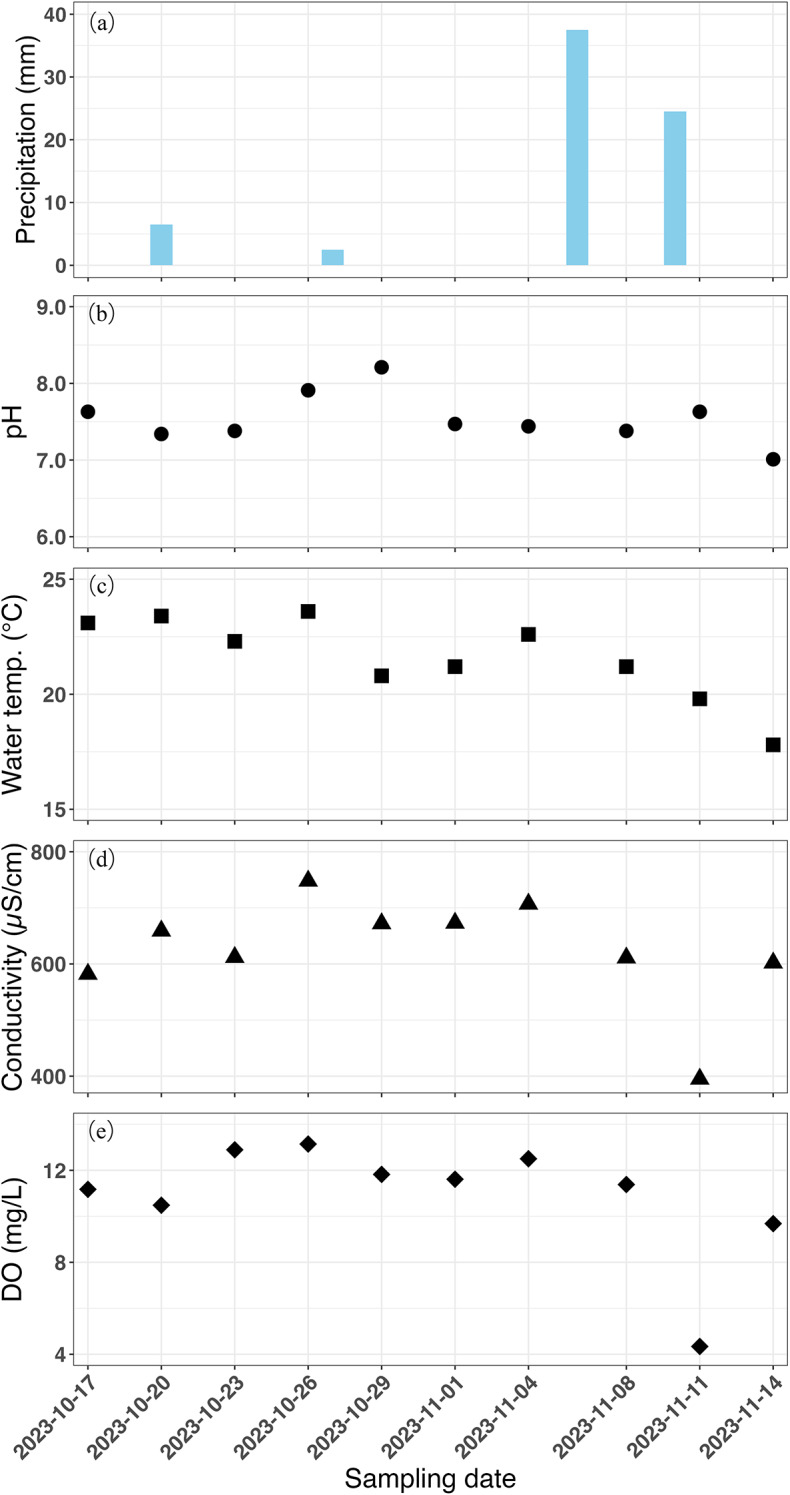
Temporal variation in (**a**) daily precipitation recorded at the meteorological observation station and water quality parameters measured at the Hirano River sampling site: (**b**) pH, (**c**) water temperature, (**d**) conductivity, and (**e**) dissolved oxygen (DO). The meteorological observation station was located approximately 6 km from the sampling site.

Figure 1 and Table S1 present precipitation data and water quality parameters measured at the Hirano River during the study period. Rainfall events were recorded on October 20, October 27, November 6, and November 10, 2023. Overall, water quality parameters remained relatively stable, except for a pronounced decline in conductivity and DO on November 11, 2023. The predefined rainfall intensity index values (see Methods) for rainfall events recorded on November 6 and November 10, 2023, were 156.2 and 10.0, respectively, when reported to one decimal place. For the November 10 event, the unrounded index value exceeded the predefined threshold of 10. Therefore, both events were classified as strong rainfall events with the potential to trigger CSOs. On November 8, 2023, sampling was conducted at 33 h after rainfall, while on November 11, 2023, sampling was conducted 15 h after rainfall.

**Fig. 2:**
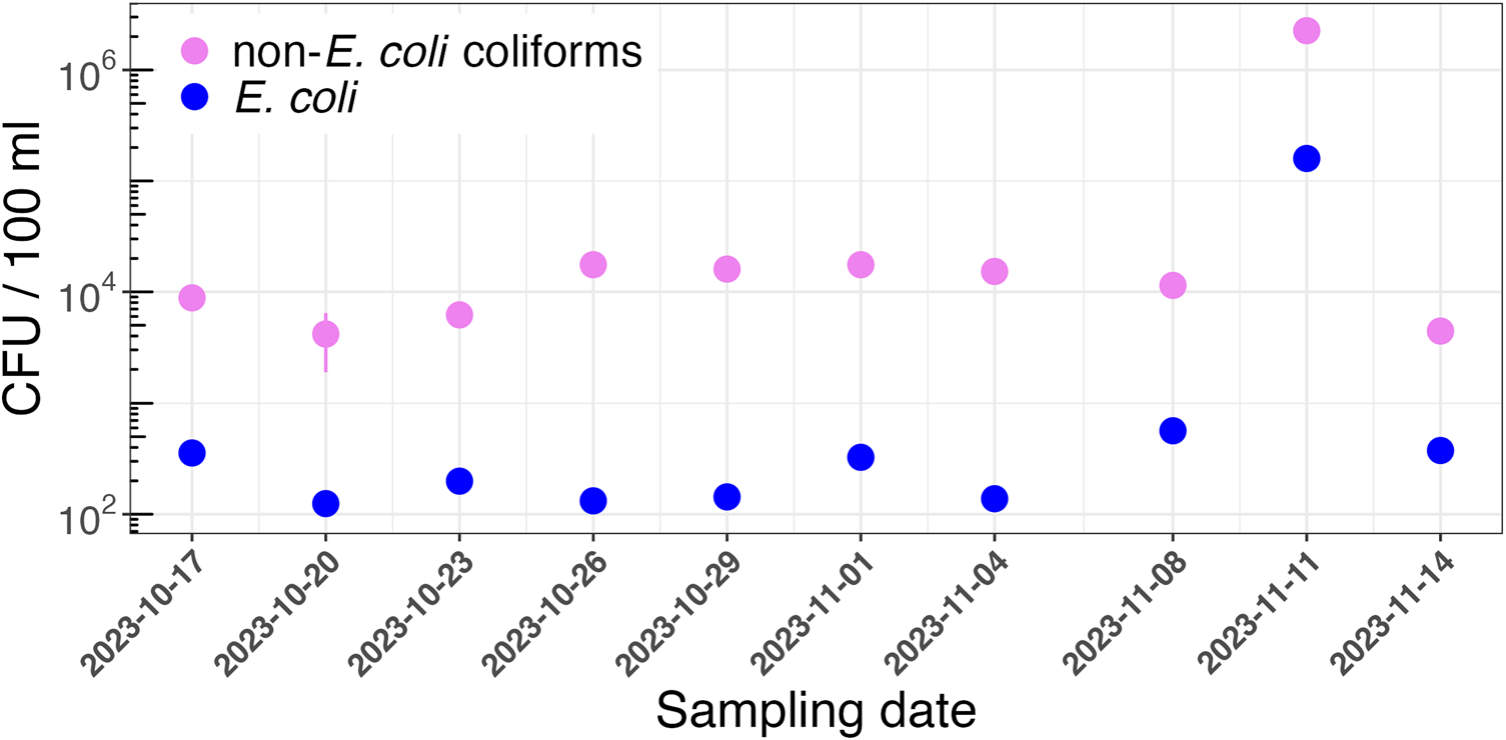
Temporal variation in colony-forming units (CFUs) of non-*E. coli* coliforms (mauve) and *Escherichia coli* (blue) at the Hirano River sampling site. Data are mean concentrations based on three technical replicates. Error bars, which are very small compared to the data points, indicate standard deviation from the mean of three technical replicates.

On November 11, 2023, *E*. *coli* and non-*E. coli* coliform concentrations in the river increased markedly compared to November 8, 2023 (Fig. 2). non-*E. coli* coliform concentrations increased from 1.14 ± 0.20 × 10⁴ CFU/100 mL on November 8, 2023, to 2.26 ± 0.18 × 10⁶ CFU/100 mL on November 11, 2023. Similarly, *E. coli* concentrations increased from 8.00 ± 0.87 × 10² CFU/100 mL to 1.59 ± 0.12 × 10⁵ CFU/100 mL over the same period, suggesting a substantial increase in the measured indicator bacterial populations following the rainfall event, potentially associated with CSO-related inputs. During this period, relatively lower conductivity compared to typical conditions in this river system and low DO conditions were observed. Similar patterns have been reported elsewhere: in the Seine River, *E*. *coli* and intestinal enterococci concentrations increased by two orders of magnitude following CSO events^27^, and McGinnis et al.^28^ reported significant correlations between human *Bacteroides*, total coliforms, and recent CSO and rainfall events.

**Fig. 3:**
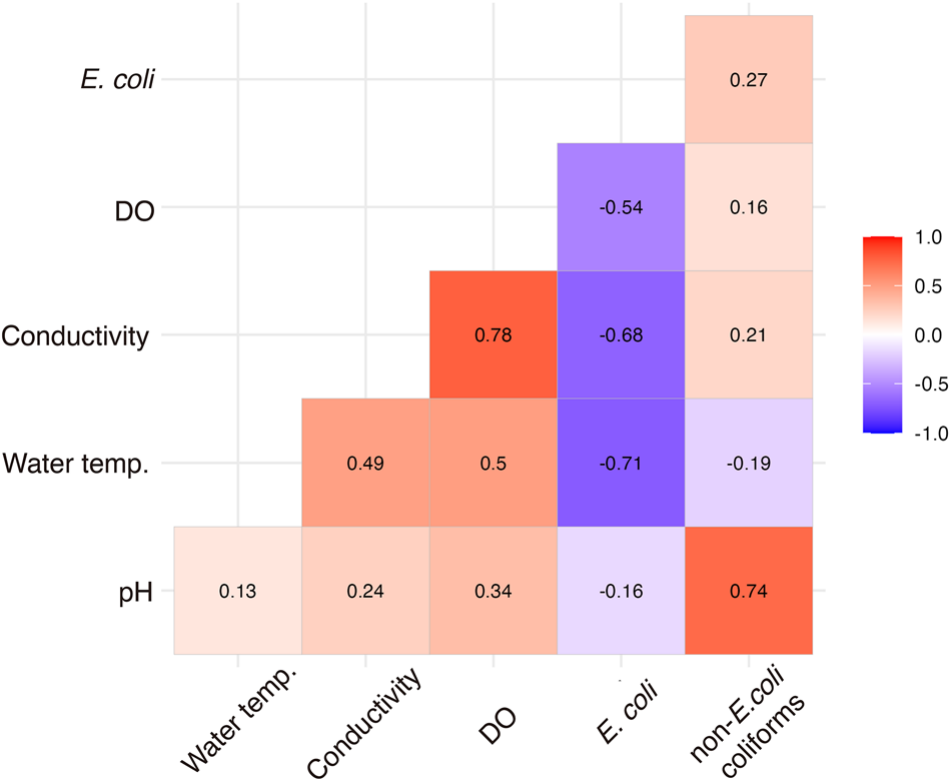
Spearman correlation matrix showing relationships between environmental parameters and bacterial indicators. Positive correlations are shown in red and negative correlations in blue, with color intensity proportional to correlation strength. Only the lower triangle of the matrix is displayed for clarity.

During CSO events, untreated sewage can introduce FIB along with dissolved ions and organic matter into receiving waters. Electrical conductivity should therefore be interpreted primarily as an indicator of changes in ionic composition^29^, rather than as a direct measure of bacterial or organic matter concentrations. Previous studies have also demonstrated that conductivity is associated with variations in the fluorescence components of dissolved organic matter (DOM), reflecting relationships between ionic strength and DOM characteristics^30^. In urban rivers, conductivity has been reported to correlate with FIB concentrations and can serve as a rapid screening indicator of microbial contamination, although this relationship is site-specific^31^. However, during CSO events, conductivity does not necessarily increase monotonically; instead, it may decrease as stormwater input increases and the sewage fraction becomes diluted^32^. The decline in conductivity observed on November 11, 2023, may therefore reflect this dilution process. Despite the reduced conductivity, *E*. *coli* and non-*E. coli* coliform concentrations were two orders of magnitude higher than during dry-weather conditions. As noted in previous studies, conductivity may not serve as a consistent indicator of FIB abundance during CSO events^27,33^. In the present dataset, *E. coli* concentrations showed negative Spearman correlations with conductivity (ρ = −0.68) and DO (ρ = −0.54), whereas non-*E. coli* coliform concentrations showed weak positive correlations with conductivity (ρ = 0.21) and DO (ρ = 0.16) (Fig. 3). Thus, although *E. coli* and non-*E. coli* coliforms exhibited similar temporal patterns (Fig. 2), their associations with conductivity and DO differ in direction and magnitude in this dataset. Given the limited number of sampling dates (*n* = 10) and the absence of statistical significance testing, these correlations should be interpreted descriptively and do not establish different environmental controls on the two bacterial groups.

Both non-*E. coli* coliforms and *E*. *coli* were consistently detected in the river, even during dry weather. Except for the wet-weather sample collected on November 11, 2023, following a strong rainfall event classified as having the potential to trigger a CSO, non-*E. coli* coliform concentrations ranged between 3.0 × 10^3^ and 2.0 × 10^4^ CFU/100 mL, while *E*. *coli* concentrations ranged between 1.0 × 10^2^ and 5.0 × 10^2^ CFU/100 mL (Fig. 2). A GLMM applied to log_10_-transformed CFU data indicated that *E*. *coli* concentrations differed significantly from non-*E. coli* coliform concentrations (estimate = − 1.58, 95% CI − 1.69 to − 1.48, *P* < 0.001), being approximately 30 to 50 times lower on average. Thus, *E*. *coli* concentrations were approximately 2–3% of those of the non-*E. coli* coliform fraction in the Hirano River. Geomorphologically, the river belongs to the Yamato River system. Using the same CHROMagar™ ECC medium, Uranishi et al. ^34^ detected coliform levels ranging between 1.0 × 10^2^ and 1.0 × 10^5^ MPN/100 mL and *E*. *coli* levels between 1.0 × 10^1^ and 2.0 × 10^3^ MPN/100 mL in the Yamato River, based on monthly sampling conducted throughout 2020 (12 sampling events). These concentrations were similar to those observed previously in the same river system.

The non-*E. coli* coliforms include various environmental species such as *Klebsiella*, *Enterobacter*, and *Citrobacter*^35^, so their detection in river water is unsurprising. Although WHO guidelines on recreational water quality emphasize *E*. *coli* for its high specificity as an indicator^36^, recent studies have documented its persistence and even naturalization in the environment independent of warm-blooded hosts^8,37^. A review by Devane et al.^38^ examined the phenomenon of naturalized FIB, which can persist and potentially multiply in environmental reservoirs like stream sediments. Another review^39^ provided a comprehensive synthesis of research conducted over 3 decades on the decay and persistence of fecal microbiota within aquatic habitats. Our consistent detection of *E. coli* under both dry and wet weather conditions supports these observations and suggests a chronic environmental presence rather than occurrence only after rainfall events potentially associated with CSO-related inputs.

**Fig. 4:**
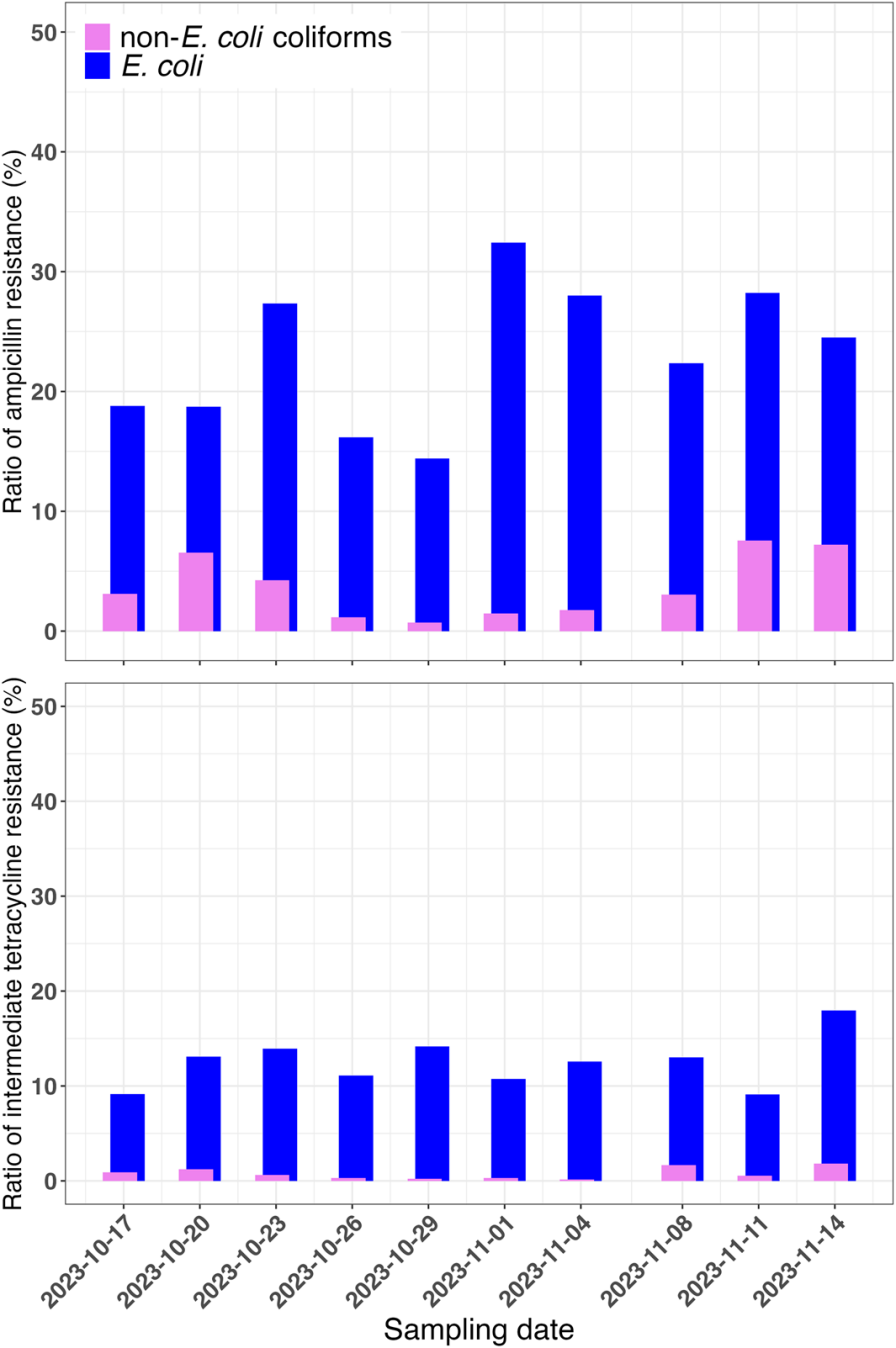
Temporal variation in (top) ampicillin resistance and (bottom) intermediate tetracycline resistance rates among non-*E. coli* coliforms and *E. coli* during the sampling period. Resistance rates (%) were calculated from mean CFU values derived from three technical replicates for each sampling date. Because replicate-level colony counts on antibiotic-supplemented and unsupplemented media were not paired for ratio calculation, error bars are not shown.

Coliforms, particularly *E*. *coli*, play central roles in the acquisition, maintenance, and dissemination of antibiotic resistance genes^40^. The high resistance ratios observed among other coliforms in clinical surveillance, such as *K*. *pneumoniae* and *E*. *cloacae*, suggest that it may also be important to monitor these species for AMR potential in environmental settings^16^. Under the One Health framework, identifying major AMR carriers underscores the interconnectedness of human, animal, and environmental reservoirs. Although *K. pneumoniae* and *E. cloacae* exhibit broad environmental distributions, quantitative data on their proportions within total coliforms are limited^41^. Therefore, to compare antibiotic resistance within the culturable coliform groups measured in this study, we assessed ampicillin resistance and intermediate tetracycline resistance in *E. coli* and the non-*E. coli* coliform fraction differentiated on CHROMagar™ ECC medium (Fig. 4). *Escherichia coli* exhibited markedly higher resistance ratios, averaging 23.1% for ampicillin resistance and 12.3% for intermediate tetracycline resistance, compared with 3.7% and 0.8%, respectively, in the non-*E. coli* coliform fraction represented by mauve colonies on CHROMagar™ ECC medium.

Exploratory outlier screening of the maximum resistance ratio in each time series did not identify any value exceeding the IQR-based upper bound (Table S2). Given the small number of sampling dates (*n* = 10), this result was used only to describe temporal variability. Three descriptive observations regarding the resistance ratios are presented below.

First, ampicillin resistance was more prevalent than intermediate tetracycline resistance in both groups. A previous study of the Yasu and Hino rivers in Japan similarly reported higher rates of ampicillin-resistant *E*. *coli* (average 25%) compared to tetracycline resistance (0–12%)^42^, consistent with previous environmental and animal surveillance reports, while clinical data on tetracycline resistance in *E. coli* remain limited^16^. In contrast, *E. coli* isolates from river and drinking water sources in Hangzhou, China, showed greater tetracycline resistance (42%) than ampicillin resistance (29%)^43^, highlighting regional variation driven by antibiotic usage and environmental conditions. However, differences in resistance definitions among studies should be considered.

Second, although bacterial concentrations increased by more than two orders of magnitude on November 11, 2023, following a strong rainfall event classified as having the potential to trigger a CSO, the resistance ratios did not show an unusually large increase. Untreated sewage can contain high concentrations of *E*. *coli* and antibiotic-resistant bacteria and may contribute to their increased abundance in receiving waters^44,45^. However, because CSO discharge was not directly monitored at the time of sampling and the source of fecal contamination was not confirmed, the contribution of CSO-related inputs to the observed bacterial concentrations and resistance ratios could not be determined. Future monitoring efforts may benefit from incorporating a broader range of clinically relevant, human-specific antimicrobials to distinguish better AMR signatures linked to CSO contributions more clearly.

Third, *E*. *coli* exhibited higher resistance rates than observed in the non-*E. coli* coliform population, which comprises a mixture of multiple coliform taxa. However, because *E. coli* concentrations were only approximately 2–3% of those of the non-*E. coli* coliform fraction, their contribution to the absolute concentration of antibiotic-resistant coliform colonies was limited, and the taxonomic composition of resistance within the non-*E. coli* coliform fraction remains unresolved.

Although *E. coli* represented only a minor fraction of the enumerated coliform population in the Hirano River, it consistently exhibited higher resistance ratios than the non-*E. coli* coliform fraction. In contrast, because the non-*E. coli* coliform fraction occurred at substantially higher concentrations, it accounted for a greater absolute concentration of antibiotic-resistant coliform colonies. These results suggest that *E. coli* may be useful as an indicator of elevated resistance ratios among culturable coliforms^46^, whereas the non-*E. coli* coliform fraction contributed more substantially to the absolute concentration of antibiotic-resistant coliform colonies. Thus, these two operationally defined coliform groups provide complementary information for evaluating antibiotic resistance in urban river water.

A limitation of this study is that non-*E. coli* coliforms were analyzed as a composite group. Therefore, species-specific comparisons of resistance profiles of individual taxa such as *Klebsiella* and *Enterobacter* could not be performed. The observed range of ampicillin resistance ratios in *E*. *coli* (16.2–32.4%) was slightly lower than hospital-associated levels (50.8% in 2023)^16^, but consistent with other environmental reports. For example, a previous study^42^ reported an average ampicillin resistance rate of 25.2% and tetracycline resistance rates of 0–12.1% in *E. coli* isolated from 2 major rivers flowing into Lake Biwa, Japan, based on samples collected under dry weather conditions from December 2019 to July 2020. In the present study, a threshold of 10 µg/mL was used to define intermediate tetracycline resistance, whereas previous studies have generally defined resistance based on clinical breakpoint criteria. Given the differences in resistance breakpoints and the limited duration of our study, direct comparisons may not be appropriate. These observations suggest that *E*. *coli* is an important contributor to environmental AMR. Therefore, increases in its abundance may facilitate further dissemination of AMR in aquatic ecosystems. Future studies should incorporate fecal source tracking approaches using qPCR-based microbial source tracking markers to better identify contamination sources and distinguish human-derived fecal inputs from those derived from domestic animals or wildlife, a distinction that could not be made in the present study^47^. In addition, genomic approaches, such as whole-genome sequencing of colony isolates or shotgun metagenomic analysis, would enable more detailed characterization of the taxonomic composition of the non-*E. coli* coliform fraction represented by mauve colonies on CHROMagar™ ECC medium, thereby improving the interpretation of AMR dynamics in heterogeneous coliform communities^48,49^.

## Conclusion

Following a strong rainfall event classified as having the potential to trigger a CSO, non-*E. coli* coliform and *E. coli* concentrations increased by more than 100-fold. This temporal association with rainfall may reflect CSO-related inputs as well as other sources of fecal contamination, such as runoff from domestic animals or wildlife, but the relative contributions of these sources could not be determined in the present study.

Despite these increases in *E. coli* and non-*E. coli* coliform concentrations, the measured ampicillin resistance and intermediate tetracycline resistance ratios did not increase following rainfall events. The consistent observation of AMR in *E. coli* under dry and wet weather conditions suggests the persistent presence of AMR *E. coli* in the river. In the context of this study, wet weather conditions were defined as those following rainfall events classified as having the potential to trigger CSOs, whereas dry weather conditions were defined as those not preceded by recent rainfall. Although *E. coli* concentrations were only approximately 2–3% of those of the non-*E. coli* coliform fraction, *E. coli* exhibited higher antibiotic resistance ratios, suggesting that it is a sensitive indicator of AMR. In contrast, the non-*E. coli* coliform fraction accounted for a greater absolute concentration of antibiotic-resistant coliform colonies.

Environmental parameters influencing *E. coli* dynamics may differ from those affecting other coliform species. While the route of chronic contamination remains unclear, identifying its sources and understanding *E. coli* behavior in urban rivers is critical for mitigating the spread of environmental AMR under the One Health framework. These goals may be achieved through broadening AMR profiles and conducting genomic analysis to determine whether AMR is associated with transferable genetic elements.

## Supplementary Information

Below is the link to the electronic supplementary material.

Supplementary Material 1.

## Data Availability

The datasets generated during and/or analyzed during the current study are available from the corresponding author on reasonable request.
