# Supplementary material for: The potential role of Escherichia coli as an indicator of environmental antimicrobial resistance in an urban river in Japan
Source: Sci Rep. 2026 Jun 28;16:25501. doi: 10.1038/s41598-026-56659-3 (PMC13476236; doi:10.1038/s41598-026-56659-3)
Supplement: Supplementary file 1 [file 41598_2026_56659_MOESM1_ESM.docx]

**Fig. S1.** Aerial photograph of the study site, taken by the Geospatial Information Authority of Japan (GSI) in 2006.　The combined sewer overflow (CSO) outlet is located approximately 100 m upstream of the sampling point. River water flows from the upstream CSO outlet toward the downstream sampling point.
